# Risk factor analysis and nomogram for predicting massive postoperative gastrointestinal bleeding in patients with Crohn’s disease: a multicenter retrospective study

**DOI:** 10.3389/fmed.2025.1586969

**Published:** 2025-08-06

**Authors:** Mengyi Zhang, Danhua Yao, Yuhua Huang, Qi He, Yining He, Yiran Jiao, Yuanruohan Zhang, Chunqiu Chen, Zhe Cui, Yousheng Li

**Affiliations:** ^1^Department of General Surgery, Shanghai Ninth People’s Hospital, Shanghai Jiao Tong University School of Medicine, Shanghai, China; ^2^Biostatistics Office of Clinical Research Unit, Shanghai Ninth People’s Hospital, Shanghai Jiao Tong University School of Medicine, Shanghai, China; ^3^Department of Abdominal Surgery, Shanghai Tenth People’s Hospital, Tongji University School of Medicine, Shanghai, China; ^4^Department of Gastrointestinal Surgery, Renji Hospital, Shanghai Jiao Tong University School of Medicine, Shanghai, China

**Keywords:** Crohn’s disease, gastrointestinal bleeding, postoperative complication, nomogram, risk factors

## Abstract

**Background:**

Massive postoperative gastrointestinal bleeding is a severe postoperative complication of Crohn’s disease (CD) with a high mortality rate, and deteriorating patients’ recovery. However, there are few related studies, and it lacks effective prevention measures. Therefore, we conducted a multicenter study to explore the risk factors for massive postoperative gastrointestinal bleeding in CD patients.

**Methods:**

This study was a multicenter retrospective case-control study. Patients who were diagnosed with CD and underwent gastrointestinal (GI) surgery were enrolled. The control group was matched 1:4 for gender and age. By comparing perioperative medical information between two groups, risk factors were identified through logistic regression analysis. A nomogram was constructed and internal validation was performed by bootstrap resampling.

**Results:**

A total of 170 patients were included. Multivariable logistic regression revealed the independent predictors of massive postoperative gastrointestinal bleeding involving the number of previous abdominal surgeries (OR = 2.56, 95% CI = 1.54–4.24), GI bleeding history (OR = 6.17, 95% CI = 1.59–23.97), serum albumin (ALB) (OR = 0.88, 95% CI = 0.81–0.96), and Nutrition Risk Screening 2002 (OR = 1.57, 95% CI = 1.08–2.29). The nomogram achieved an area under the curve (AUC) value of 0.85 (95% CI: 0.76–0.93). In internal validation, the AUC value was 0.976 (95% CI: 0.955–0.997). Calibration curves showed good alignment. DCA demonstrated that the diagnostic model had good clinical efficiency.

**Conclusion:**

The risk of massive postoperative gastrointestinal bleeding in CD patients will be increased with a GI bleeding history, more previous abdominal surgeries, higher nutrition risk, and lower ALB level. Our nomogram model is effective and could be a useful tool for prediction.

## Introduction

Crohn’s disease (CD) is a chronic, inflammatory bowel disorder characterized by transmural inflammation of the gastrointestinal (GI) tract, leading to a wide range of clinical manifestations and varying phenotypes of disease behavior (1). Most patients have experienced progression to complications, such as bowel obstruction, perforation, fistula and intra-abdominal abscess, therefore surgical intervention is often necessary for patients with CD. About half of CD patients undergo segmental resection surgeries in the 10 years of disease duration (2, 3), and approximately 50%–80% of patients experience surgical treatments during their lifetime (4–6). While surgery can provide relief from the uncomfortable symptoms and rescue urgent events, it also carries the risk of significant postoperative complications, including hemorrhage, anastomotic leak, ileus, and all kinds of infections. The incidence of postoperative complications ranges from 10% to 56% according to literatures (7–11).

Among various complications, postoperative GI bleeding is a common complication for CD patients who underwent bowel surgeries, ranging from 1% to 9.2% (10, 12–17). Especially, massive postoperative GI bleeding is one of the major causes of death, despite having an uncommon rate. It usually results in prolonged hospitalization, and increased morbidity of surgical complications, including infections, organ dysfunctions, and functional declines, as well as mortality (14, 17, 18). Currently, the commonly used treatments for massive postoperative GI bleeding are limited, such as hemostatic medication, glucocorticoids, and endoscopy (18). Emergency surgical operation is often the last way to stop bleeding, but the therapeutic effect is uncertain. Despite the clinical significance, few studies focused on this topic. On one hand, there is a lack of consensus in the definition of massive postoperative GI bleeding. On the other hand, methods for prediction and effective prevention measures are limited, but of great significance for clinical work.

To address these issues, we conducted a multicenter, retrospective, 1:4 matched case–control study reviewing 170 CD patients who underwent bowel surgery, aiming to identify the key risk factors associated with massive postoperative GI bleeding and develop a predictive model for early recognition of high-risk patients.

## Materials and methods

### Definitions

Massive GI bleeding is defined as (a) hemodynamic impairment exists and active fluid resuscitation is required. At least one of the following requirements is met: heart rate > 120 bpm; systolic blood pressure < 90 mmHg; skin or conjunctival paleness; capillary refill > 3 s; ≥ 2 comorbidities including cardiovascular disease, cirrhosis, renal disease, diabetes mellitus, and malignancy; (b) hemoglobin concentration is decreasing and blood transfusion or surgical treatment is required. At least one of the following requirements is met: hemoglobin concentration less than 80 g/L or reduced at least 20 g/L from baseline; red blood cell transfusion greater than four units; blood loss greater than 50% of total blood volume; surgical hemostasis is necessary according to the doctor’s assessment; (c) with symptoms of gastrointestinal bleeding (18–20). All of (a), (b), and (c) should be simultaneously satisfied.

### Study design

We performed a multicenter, retrospective, 1:4 matched case–control study involving three hospitals in Shanghai, China, including Shanghai Jiao Tong University School of Medicine Affiliated Ninth People’s Hospital (center 1), Shanghai Jiao Tong University School of Medicine Affiliated Renji Hospital (center 2), and Shanghai Tenth People’s Hospital of Tongji University (center 3). Clinical data of patients with CD who underwent bowel surgeries between August 2017 and February 2024 were retrospectively reviewed in this study. Inclusion criteria are: patients diagnosed with CD; aged over 16 years old; and who underwent intestinal surgery. Patients with preoperative gastrointestinal bleeding were excluded. All patients with massive postoperative GI bleeding were enrolled as the case group. For each case, four CD patients without massive postoperative GI bleeding were selected as control, matched by gender and age through randomly individual matching without replacement. Specifically, the patients of control group were selected as follows. First, exact matching for gender was applied. Then, among each gender, four controls were matched to one case by age (± 3 years). To minimize selection bias, the same individual can be sampled only once. Each disease case was then checked for finding sufficient number of controls.

Ethical approval was obtained from the Institutional Review Board of Shanghai Ninth People’s Hospital (No. SH9H-2024-T140-1) and was performed in accordance with the ethical standards laid down in the 1964 Declaration of Helsinki and its later amendments. All participants provided written informed consent to allow the use of their anonymized clinical data for research purposes.

### Data collection

All demographic, clinical and laboratory details were extracted from the medical records. Montreal classification (21), Crohn’s Disease Activity Index (CDAI) (22), and Nutrition Risk Screening 2002 (23), venous thromboembolism (VTE) risk assessment with Caprini score (24) were assessed on the admission date. For laboratory data, those tested on the last day before surgery were extracted. Predictive Nutritional Index (PNI) was calculated using the formula 10 × serum albumin (ALB) (g/dL) + 0.005 × total lymphocyte count (/mm^3^) (25). For medical history, patients’ previous diagnoses such as intestinal fistula, abdominal abscess, intestinal obstruction, gastrointestinal bleeding, intestinal perforation history, hypertension, diabetes and abnormality of liver function were included in statistics, along with the number of previous abdominal surgeries (e.g., cesarean section, intestine resection, and bariatric surgery) and the course of disease before surgery. Abnormality of liver function was defined as Child–Pugh class B or C (26). For medication, we measured the exposure to biologics (e.g., adalimumab, infliximab, vedolizumab, and ustekinumab) within 12 weeks before surgery and the use of immunomodulators (e.g., methotrexate, azathioprine, and cyclosporine), aminosalicylates (ASA) (e.g., sulfasalazine, mesalamine, and olsalazine), and corticosteroids (e.g., prednisone and methylprednisolone) 1 week before surgery. Surgical procedure details were extracted from operative documents. Preoperative usage of anticoagulant drug (e.g., heparin and derivative substances, and vitamin K antagonists) and antiplatelet drugs (e.g., aspirin, clopidogrel, prasugrel, and ticagrelor) was also collected.

### Statistical analysis

The statistical analysis was performed using R software (version 4.3^1^). The distribution normality of continuous variables was assessed by the quantile-quantile plot and the Shapiro-Wilk test. Continuous variables with a normal distribution were compared by the Student’s *t*-test and those with a skewed distribution were compared by the Mann-Whitney’s U test. Categorical variables were compared by the Pearson χ^2^ test, Yates’s correction test or the Fisher’s exact test. *P*-value < 0.05 was considered as significant. The univariate logistic analysis was then applied for initial screening for the risk factors. The independent variables which shared no interrelationships and with a *P*-value < 0.1 in univariate logistic analysis were then included in multivariate analysis. If two variables were considered correlated, variable that is more clinically relevant or that can be directly measured would be selected. Multivariate logistic analysis was then applied with stepwise selection with Akaike information criterion. Only the independent risk factors with a *P*-value < 0.05 were included into the model. Odds ratios (ORs) and 95% confidence intervals (95% CI) are provided.

Significant risk factors in multivariate analysis were selected to construct a prediction nomogram. The model was internally validated by using 1,000 bootstrap resamples. The receiver operating characteristic (ROC) curve was undertaken and the area under the ROC curve (AUC) was used to evaluate the discrimination accuracy of the model. The calibration curve was used to evaluate predictive ability of the model, and the Hosmer-Lemeshow test was performed to evaluate the goodness of fit of the model. Clinical value of the model was estimated by decision curve analysis (DCA) (27).

## Results

**FIGURE 1:**
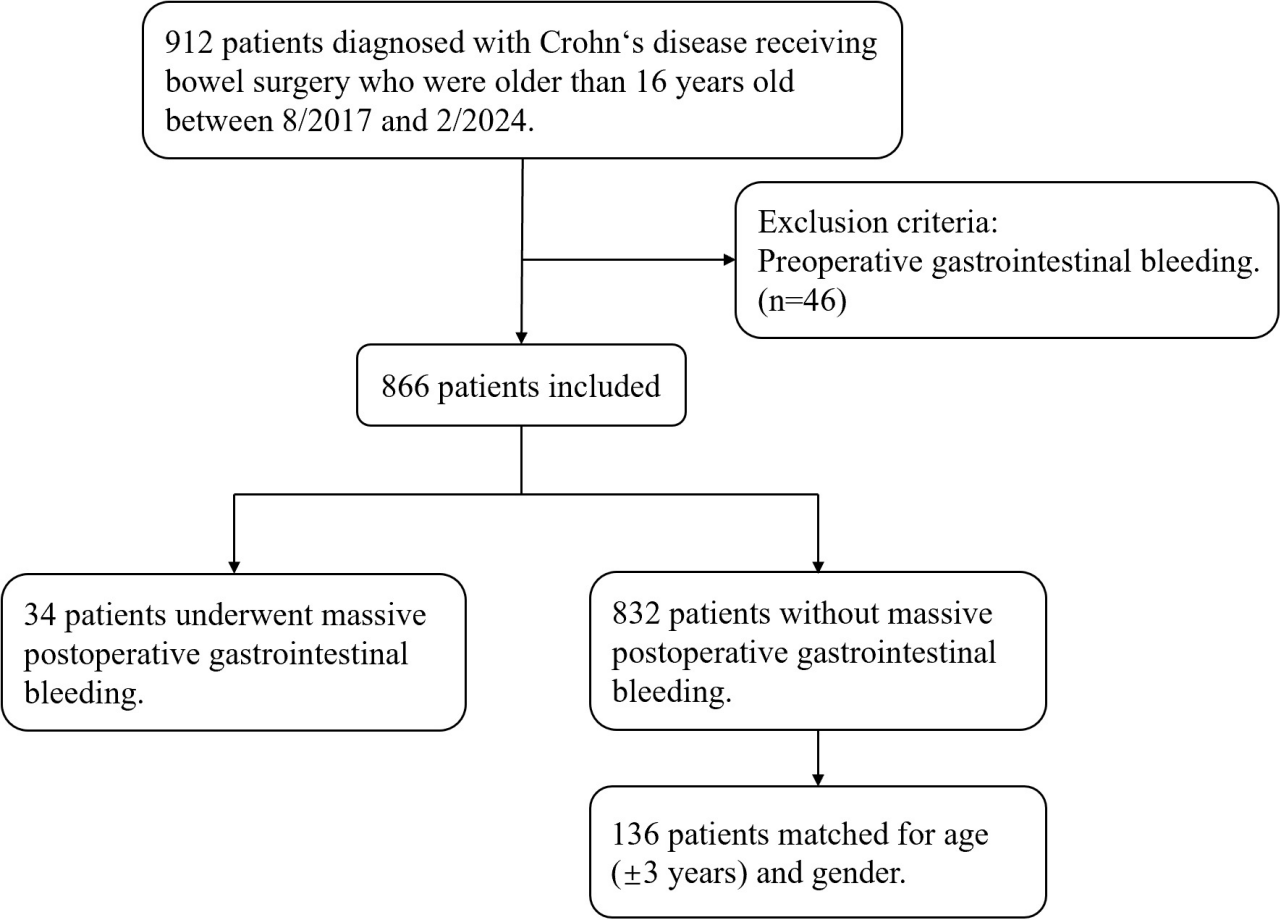
Flow chart of the patients included in the study.

During the study period, there are 866 patients diagnosed with Crohn’s disease receiving bowel surgery who are older than 16 years old and didn’t experience preoperative gastrointestinal bleeding. A total of 34 patients underwent massive postoperative gastrointestinal bleeding were included in the case group, while 136 patients, individually matched for age (± 3 years) and gender were included in the control group (Figure 1). Among 34 massive postoperative GI bleeding patients, six of them underwent secondary operations for hemostasis, and two eventually died of massive bleeding, with hospital stays ranging from 21 to 82 days. There was no recurrent hemorrhage within 90 days after discharge.

Table 1 shows the demographic and clinical characteristics of the patients in both the case and control groups. 14.71% of the patients were female in both groups, with a mean age of 41.00 ± 12.73. No difference was observed in gender, age, body mass index (BMI) and smoking condition between the cases and controls. As for the Montreal classification, no difference was observed in age, disease location and disease behavior between the two groups. The majority of patients were between 17 and 40 years old (A2) (55.88% vs. 52.94%, *P* = 0.758), had both ileal and colonic CD (L3) (67.65% vs. 52.21%, *P* = 0.105), and had a penetrating phenotype (B3) (61.76% vs. 52.94%, *P* = 0.423). 10% of the patients have upper GI disease (L4) (8.82% vs. 10.29%, *P* = 0.798), while 36.47% have perianal diseases (44.12% vs. 34.56%, *P* = 0.300). Patients with massive postoperative GI bleeding have more previous abdominal surgeries [1 (0, 2) vs. 0 (0, 1), *P* < 0.001] and longer courses of disease before surgery [10.00 (6.00, 12.75) vs. 4.50 (1.00, 9.00), *P* < 0.001]. Besides, patients with a GI bleeding history were more likely to have massive postoperative GI bleeding (29.41% vs. 3.68%, *P* < 0.001).

**TABLE 1:** Baseline demographic and clinical characteristic.

Characteristic	Total (*n* = 170)	Case group (*n* = 34)	Control group (*n* = 136)	*P*-value
Gender (female)	25 (14.71)	5 (14.71)	20 (14.71)	1.000
Age (years)	41.00 ± 12.73	41.24 ± 12.78	40.94 ± 12.76	0.905
BMI (kg/m^2^)	19.08 ± 3.04	18.30 ± 2.92	19.28 ± 3.05	0.093
Active smoking	7 (4.12)	2 (5.88)	5 (3.68)	0.563
**Montreal classification**				
A1 (≤ 16)	0 (0.00)	0 (0.00)	0 (0.00)	0.758
A2 (17–40)	91 (53.53)	19 (55.88)	72 (52.94)	
A3 (> 40)	79 (46.47)	15 (44.12)	64 (47.06)	
L1 (ileal)	56 (32.94)	6 (17.65)	50 (36.76)	0.105
L2 (colonic)	20 (11.76)	5 (14.71)	15 (11.03)	
L3 (ileo-colonic)	94 (55.29)	23 (67.65)	71 (52.21)	
L4 (upper GI)	17 (10.00)	3 (8.82)	14 (10.29)	0.798
B1 (inflammatory)	27 (15.88)	3 (8.82)	24 (17.65)	0.423
B2 (stricturing)	50 (29.41)	10 (29.41)	40 (29.41)	
B3 (penetrating)	93 (54.71)	21 (61.76)	72 (52.94)	
Perianal disease	62 (36.47)	15 (44.12)	47 (34.56)	0.300
**Medical history**				
Number of previous abdominal surgeries	0 (0, 1)	1 (0, 2)	0 (0, 1)	< 0.001*
Course of disease before surgery (years)	6.00 (2.00, 10.00)	10.00 (6.00, 12.75)	4.50 (1.00, 9.00)	< 0.001*
Intestinal fistula history	82 (48.24)	20 (58.82)	62 (45.59)	0.167
Abdominal abscess history	42 (24.71)	10 (29.41)	32 (23.53)	0.477
Intestinal obstruction history	81 (47.65)	16 (47.06)	65 (47.79)	0.939
GI bleeding history	15 (8.82)	10 (29.41)	5 (3.68)	< 0.001*
Intestinal perforation history	60 (35.29)	12 (35.29)	48 (35.29)	1.000
Hypertension	7 (4.12)	1 (2.94)	6 (4.41)	0.700
Diabetes	2 (1.18)	1 (2.94)	1 (0.74)	0.286
Abnormality of liver function	5 (2.94)	3 (8.82)	2 (1.47)	0.023
**Medication history**				
5-ASA	13 (7.65)	4 (11.76)	9 (6.62)	0.312
Immunomodulators	13 (7.65)	3 (8.82)	10 (7.35	0.773
Azathioprine	10 (5.88)	2 (5.88)	8 (5.88)	1.000
Methotrexate	3 (1.76)	1 (2.94)	2 (1.47)	0.490
Biologics	50 (29.41)	9 (26.47)	41 (30.15)	0.674
Infliximab	27 (15.88)	4 (11.76)	23 (16.91)	0.463
Vedolizumab	10 (5.88)	2 (5.88)	8 (5.88)	1.000
Ustekinumab	8 (4.71)	2 (5.88)	6 (4.41)	1.000
Adalimumab	5 (2.94)	1 (2.94)	4 (2.94)	1.000
Corticosteroids	8 (4.71)	2 (5.88)	6 (4.41)	0.717
**Preoperative evaluation and treatment**				
HGB (g/L)	112.36 ± 22.67	105.18 ± 27.54	114.15 ± 21.02	0.083
PLT (× 10^∧^9/L)	239.00 (196.25, 310.75)	228.00 (154.75, 267.75)	244.00 (199.00, 311.25)	0.088
CRP (mg/L)	4.79 (1.94, 23.85)	11.90 (1.93, 40.00)	4.50 (1.94, 21.52)	0.186
TT (sec)	16.94 ± 1.80	17.23 ± 2.83	16.87 ± 1.44	0.474
INR	1.06 ± 0.12	1.12 ± 0.12	1.05 ± 0.12	0.002
D-dimer (mg/L)	0.46 (0.26, 0.79)	0.49 (0.26, 0.68)	0.43 (0.25, 0.83)	0.899
ALB (g/L)	36.79 ± 5.95	33.70 ± 5.80	37.58 ± 5.74	0.001
CDAI	252.12 ± 88.79	252.11 ± 90.80	252.12 ± 88.68	0.9990
NRS2002	2.22 ± 1.38	2.85 ± 1.37	2.06 ± 1.34	0.002
PNI	43.72 ± 7.45	40.22 ± 7.90	44.55 ± 7.12	0.003
VTE risk assessment (Caprini score)				0.010
Very low risk (0)	98 (57.65)	17 (50.00)	81 (59.56)	
Low risk (1–2)	23 (13.53)	6 (17.65)	17 (12.50)	
Moderate (3–4)	42 (24.71)	6 (17.65)	36 (26.47)	
High risk (≥ 5)	7 (4.12)	5 (14.71)	2 (1.47)	
Usage of anticoagulant drugs	6 (3.53)	5 (14.71)	1 (0.74)	< 0.001*
None	164 (96.47)	29 (85.29)	135 (99.26)	0.001
Heparin and derivative substances	2 (1.18)	2 (5.88)	0 (0.00)	
Vitamin K antagonists	4 (2.35)	3 (8.82)	1 (0.74)	
Usage of antiplatelet drugs	2 (1.18)	1 (2.94)	1 (0.74)	0.361
**Surgical procedures**				
Elective surgery (vs. emergency)	162 (95.29)	30 (88.24)	132 (97.06)	0.085
Laparoscopic surgery (vs. open)	66 (38.82)	5 (14.71)	61 (44.85)	0.001
Length of the intestine resected (cm)	15.00 (10.00, 25.00)	15.00 (10.00, 24.75)	16.00 (10.50, 25.00)	0.327
Segment of intestine resected				0.158
Small bowel	90 (52.94)	13 (38.24)	77 (56.62)	
Ileocecal	14 (8.24)	5 (14.71)	9 (6.62)	
Colon	22 (12.94)	6 (17.65)	16 (11.76)	
Small bowel and colon	44 (25.88)	10 (29.41)	34 (25.00)	

*P-value < 0.001. Data are presented as mean ± SD, number (%) or Median (1st Quartile, 3rd Quartile). BMI, body mass index; GI, gastrointestinal; ASA, aminosalicylate; HGB, hemoglobin; PLT, platelet; CRP, C-reactive protein; TT, thrombin time; INR, international normalized ratio; ALB, albumin; CDAI, Crohn’s Disease activity index; NRS2002, nutrition risk screening 2002; PNI, predictive nutritional index; VTE, venous thromboembolism.

Regarding medication, there were no statistically significant differences according to ASA, immunomodulators, biologics, and corticosteroids. Both groups showed similar proportions of medication administration, with a quarter of the patients receiving biologics (26.47% vs. 30.15%, *P* = 0.674), few patients receiving ASA (11.76% vs. 6.62%, *P* = 0.312), immunomodulators (8.82% vs. 7.35%, *P* = 0.773), and corticosteroids (5.88% vs. 4.41%, *P* = 0.717) before surgery (Table 1).

In terms of preoperative evaluation, patients with massive postoperative GI bleeding tended to have a higher INR (1.12 ± 0.12 vs. 1.05 ± 0.12, *P* = 0.002), lower ALB (33.70 ± 5.80 vs. 37.58 ± 5.74, *P* = 0.001), higher Nutrition Risk Screening 2002 score (2.85 ± 1.37 vs. 2.06 ± 1.34, *P* = 0.002), lower PNI (40.22 ± 7.90 vs. 44.55 ± 7.12, *P* = 0.003), more risk of VTE (high risk according to the Caprini score: 14.71% vs. 1.47%, *P* = 0.010), and more usage of anticoagulant drugs (14.71% vs. 0.74%, *P* < 0.001) (Table 1).

As for surgical procedures, no statistically significant differences were found in surgical type (elective vs. emergency surgery), length of the intestine resected, and segment of intestine resected. However, it is shown that patients who had an open surgery had more chance to undergo massive postoperative GI bleeding than those who had a laparoscopic surgery (14.71% vs. 44.85%, *P* = 0.001) (Table 1).

### Risk factors of massive postoperative gastrointestinal bleeding

According to the univariate analysis, significant risk factors include number of previous abdominal surgeries (OR = 2.48, 95% CI = 1.61–3.81, *P* < 0.001), course of disease before surgery (OR = 1.14, 95% CI = 1.07–1.22), *P* < 0.001), gastrointestinal bleeding history (OR = 10.92, 95% CI = 3.43–34.77, *P* < 0.001), abnormality of liver function (OR = 6.48, 95% CI = 1.04–40.47, *P* = 0.045), INRx10 (OR = 1.61, 95% CI = 1.16–2.23, *P* = 0.004), ALB (OR = 0.89, 95% CI = 0.83–0.95, *P* = 0.001), Nutrition Risk Screening 2002 (OR = 1.61, 95% CI = 1.17–2.23, *P* = 0.004), PNI (OR = 0.92, 95% CI = 0.87–0.97, *P* = 0.005), high risk of VTE (OR = 11.91, 95% CI = 2.13–66.60, *P* = 0.005), usage of anticoagulant drugs (OR = 23.28, 95% CI = 2.62–206.77, *P* = 0.005), and laparoscopic surgery (OR = 0.21, 95% CI = 0.08–0.58, *P* = 0.003).

Multivariable logistic regression identified four independent factors associated with massive postoperative GI bleeding, including the number of previous abdominal surgeries (OR = 2.56, 95% CI = 1.54–4.24, *P* < 0.001), gastrointestinal bleeding history (OR = 6.17, 95% CI = 1.59–23.97, *P* = 0.009), ALB (OR = 0.88, 95% CI = 0.81–0.96, *P* = 0.003), and Nutrition Risk Screening 2002 (OR = 1.57, 95% CI = 1.08–2.29, *P* = 0.018) (Table 2).

**TABLE 2:** Univariate and multivariate logistic regression analysis of potential risk factors for massive postoperative gastrointestinal bleeding.

Variable	Univariate analysis		Multivariate analysis	
	OR (95% CI)	*P*-value	OR (95% CI)	*P*-value
Number of previous abdominal surgeries	2.48 (1.61∼3.81)	< 0.001*	2.56 (1.54∼4.24)	< 0.001*
Course of disease before surgery (years)	1.14 (1.07∼1.22)	< 0.001*		
GI bleeding history	10.92 (3.43∼34.77)	< 0.001*	6.17 (1.59∼23.97)	0.009
Abnormality of liver function	6.48 (1.04∼40.47)	0.045		
INRx10	1.61 (1.16∼2.23)	0.004		
ALB	0.89 (0.83∼0.95)	0.001	0.88 (0.81∼0.96)	0.003
NRS2002	1.61 (1.17∼2.23)	0.004	1.57 (1.08∼2.29)	0.018
PNI	0.92 (0.87∼0.97)	0.005		
VTE risk assessment (Caprini score)				
Very low risk (0)	Reference			
Low risk (1–2)	1.68 (0.58∼4.89)	0.340		
Moderate (3–4)	0.79 (0.29∼2.18)	0.655		
High risk (≥ 5)	11.91 (2.13∼66.60)	0.005		
Usage of anticoagulant drugs	23.28 (2.62∼206.77)	0.005		
Laparoscopic surgery (vs. open)	0.21 (0.08∼0.58)	0.003		

*P-value < 0.001. OR, odds ratio; CI, confidence interval; GI, gastrointestinal; INR, international normalized ratio; ALB, albumin; NRS2002, nutrition risk screening 2002; PNI, predictive nutritional index; VTE, venous thromboembolism.

### Nomogram establishment

**FIGURE 2:**
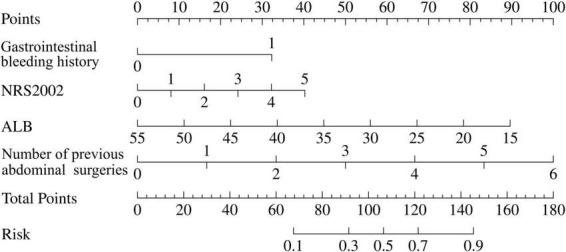
Nomogram predictive model for risk of massive postoperative gastrointestinal bleeding in Crohn’s disease (CD) patients. To calculate the risk of massive postoperative gastrointestinal bleeding, first determine the value of each factor by drawing a vertical line from raw value of the factor to the point scale. Then a total point was obtained by adding all the points of each factor. Based on the total point, draw a vertical line from total points scale to the risk scale to determine a predicted probability. The corresponding risk rate ranges from 0.1 to 0.9. The higher the total point, the greater the risk of massive postoperative gastrointestinal bleeding.

Based on the result of multivariable logistic regression analysis, a nomogram model was developed (Figure 2). Each variable was assigned a point, and a total point was calculated by summing all the points of each variable, ranging from 0 to 180 points. The corresponding risk rate ranges between 0.1 and 0.9. A higher total point indicates a greater risk of massive postoperative GI bleeding.

**FIGURE 3:**
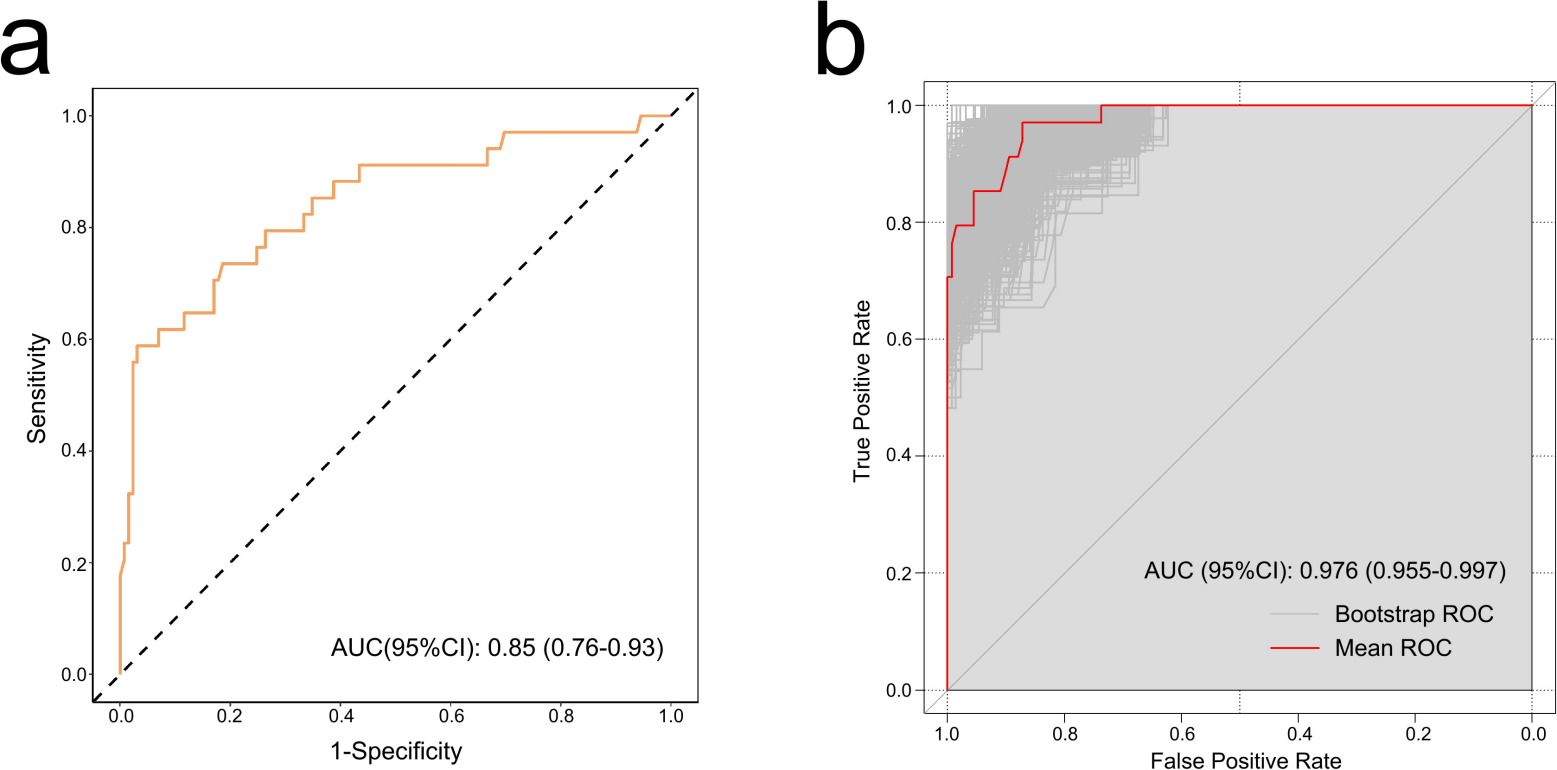
The receiver operating characteristic (ROC) curves of the nomogram. **(a)** ROC curve of the training set. Discrimination ability of this model was appraised with an area under the curve (AUC) of 0.85 (95% CI: 0.76–0.93). **(b)** ROC curve by bootstrapping for 1,000 repetitions. In bootstrap internal validation, the AUC value was 0.976 (95% CI: 0.955–0.997).

**FIGURE 4:**
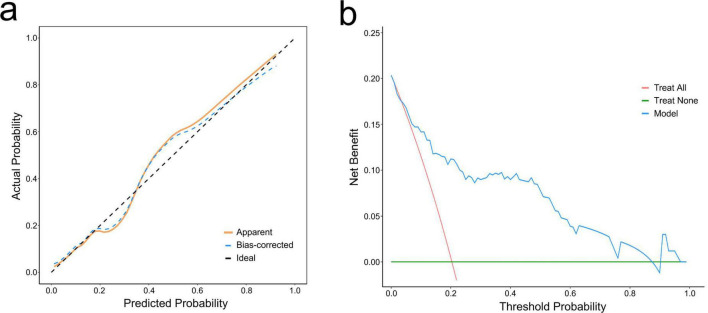
Calibration curve and decision curve analysis (DCA) of the nomogram. **(a)** Calibration curve. The dashed line represents ideal fit, where nomogram-predicted probability (x-axis) matches observed probability (y-axis). The dotted line represents actual nomogram performance. The prediction effect is more effective when it is closer to the dashed line. The solid line represents adjusted (bootstrap corrected) calibration accuracy. **(b)** DCA curve. The blue curve represents the current prediction model, the red line represents intervention for all, and the green line represents intervention for none. The decision curve showed that the threshold probability is between 3% and 88% for our nomogram.

The ROC curve was used to evaluate the accuracy of the model. The AUC of the model reached 0.85 (95% CI: 0.76–0.93) (Figure 3a). After 1,000 bootstrap internal validations, the ROC curve showed an AUC of 0.976 (95% CI: 0.955–0.997) (Figure 3b), which indicated that the model had good discrimination. A calibration curve was drawn (Figure 4a), with a good agreement between the predicted and observed probabilities. The *P*-value of the Hosmer-Lemeshow test was 0.598, also indicating that this nomogram model had excellent calibration ability. The DCA demonstrated that the nomogram had a net clinical benefit at a threshold probability between 3% and 88% (Figure 4b).

## Discussion

Massive postoperative GI bleeding as a postoperative complication in CD, is very severe and may cause death of patient. Thus, we novelly carried out this multicenter study. In this study, we identified 11 risk factors associated with massive postoperative GI bleeding in CD through univariate logistic analysis. Multivariate analysis further confirmed four independent predictors, including Nutrition Risk Screening 2002, ALB, gastrointestinal bleeding history, and the number of previous abdominal surgeries, which were incorporated into a clinically practical nomogram prediction model, with robust reliability, accuracy, and clinical utility for early identification of high-risk patients. Our findings provide a valuable tool for risk stratification and perioperative management of massive postoperative GI bleeding in CD patients.

The Nutrition Risk Screening 2002 emerged as a significant predictor, emphasizing the interplay between malnutrition risk and postoperative bleeding. Malnutrition is a common and well-documented complication in Crohn’s disease due to factors such as decreased intake, chronic inflammation and malabsorption (28). Patients with higher nutrition risk score likely exhibit catabolic states, and compromised immune function, resulting in a poor recovery from surgical trauma and vascular injury (29–31). This result supports integrating routine nutritional risk assessment into preoperative workflows, thereby decreasing the possibility of postoperative GI bleeding. Individuals with high-risk of malnutrition may benefit from perioperative nutritional supports, including total parenteral nutrition (TPN), enteral nutrition (EN), and oral nutritional supplements (ONS). Rational use and combination of different nutritional supporting method may improve patients’ nutritional status (32). Further research is needed to optimize the protocols of nutritional intervention in this population.

Preoperative albumin levels also demonstrate a significant association with GI bleeding risk. Hypoalbuminemia can reflect chronic disease activity, which may impair mucosal healing and increase vascular fragility, leading to GI bleeding. During the occurrence of IBD, inflammatory cytokines such as interleukin (IL)-6 and Tumor Necrosis Factor alpha (TNF-α) released by inflamed tissues suppress hepatic albumin synthesis (33, 34). Meanwhile, the inflammatory response increases intestinal permeability, leading to the leakage of serum albumin into the interstitial cavity (35). This results in an elevated clearance rate and reduced synthesis of serum albumin, which is why IBD patients often exhibit low serum albumin levels. It is also possible that low albumin directly leads to compromised tissue repair and coagulation factor synthesis, which cause postoperative GI hemorrhage. Future studies should explore whether albumin level directly correlates with the risk of GI bleeding or simply serves as a marker of disease severity developing secondary GI bleeding. Additionally, serum albumin can bind to drugs, thereby influencing their diffusion, transport, and elimination. Consequently, it may be associated with treatment response in IBD patients. Previous studies have indicated that low serum albumin levels are linked to increased clearance of infliximab and vedolizumab (36, 37). Poor preoperative response to biologic agents would lead to inadequate inflammation control, which may heighten the risk of postoperative complications, including massive GI bleeding.

Patients with a prior episode of GI bleeding faced elevated postoperative GI bleeding risk, suggesting an underlying predisposition to vascular or mucosal abnormality. The chronic inflammatory nature of CD can lead to vascular damage and mucosal ulceration, which may lead to further bleeding, particularly in the postoperative situation. Among those patients with previous GI bleeding history, they may have a persistent disease activity or irreversible damage to the microvasculature (38). This finding underscores the need for heightened postoperative monitoring in patients with such medical histories, as they may benefit from earlier intervention or tailored prophylactic strategies.

A history of multiple abdominal surgeries was independently associated with massive postoperative GI bleeding risk, likely reflecting the cumulative impact of tissue adhesions, altered vascular anatomy, and prolonged operative times (39, 40). Adhesions and fibrotic tissue may harbor fragile collateral vessels prone to hemorrhage. Thus, repeat surgeries necessitate technically challenging dissections, increasing the likelihood of inadvertent vascular injury or compromised anastomotic perfusion. Additionally, based on our data, 73% of previous abdominal surgeries in total patients were performed due to CD-related complications, indicating that patients with multiple prior surgeries are more likely to have more severe and complex disease conditions. These characteristics significantly enhance the difficulty of operation, thus prolonging the operation time and elevating the potential risk of massive postoperative GI bleeding, which reinforces the importance of meticulous surgical planning.

In the univariate analysis, there still existed several potential risk factors that were not included into the model because of their co-relationship with other variables and the maximum variable inclusion restriction. Elevated INR, high risk of VTE, and usage of anticoagulant drugs were found to be associated with massive postoperative GI bleeding, indicating the possible role of coagulation abnormalities in its occurrence. IBD is connected with a disturbance of blood coagulation due to the complex interplay between acquired endothelial dysfunction, abnormalities of platelets, activation of the coagulation system and impaired fibrinolysis (41). Abnormal liver function was also relevant to the disease occurrence. Liver dysfunction may be related to inflammation, malabsorption, or caused by treatments, since most of the medications used in IBD are potentially hepatotoxic. Hepatitis B reactivation during immunosuppressive therapy is also a major concern (42). Given the liver’s crucial role in the synthesis of coagulation factors and clearance of activation products, impaired hepatic function can significantly predispose patients to bleeding complications. Therefore, careful perioperative management of liver function and coagulation status is essential for minimizing the risk of massive postoperative GI bleeding.

Building upon the four independent risk factors identified in the multivariable logistic regression analysis, a nomogram-based predictive model was constructed. Unlike previous models that focus on general postoperative complications in CD patients (43, 44) or hemorrhage risk following GI surgery across diverse patient populations (45), our study is the first research specifically designed for preoperative risk stratification of massive postoperative GI bleeding in Crohn’s disease, addressing a critical unmet need in the surgical management of CD. By utilizing routinely available clinical and laboratory parameters, this visual scoring system facilitates rapid quantification of individual patient risk, allowing clinicians to implement early interventions before surgery. We expect that our model could serve as a practical and cost-effective tool for risk stratification and contribute to preventing massive postoperative GI bleeding for CD patients.

Our study combined data from 160 patients across three hospitals, and systematically evaluated various perioperative potential risk factors, enhancing the sample representativeness and result reliability. However, there remain several limitations in our study. First, the study is based on a retrospective design, thus, it cannot exclude all potential bias. Second, given the rare incidence of massive postoperative GI bleeding, the sample size of the model is relatively small. For the accuracy of the model, only internal validation was performed due to the restriction of sample size. A larger sample size with more other medical institutions joining in and an external validation would be required for further validation.

## Conclusion

The risk of massive postoperative GI bleeding in CD patients will be increased with a history of gastrointestinal bleeding, more previous abdominal surgeries, higher nutrition risk score, and lower ALB value. We construct a nomogram model, which can effectively predict the risk of massive postoperative GI bleeding in CD patients, with high discriminative ability and clinical application value.

## Data Availability

The raw data supporting the conclusions of this article will be made available by the authors, without undue reservation.

## Footnotes

1
https://www.r-project.org/
